# Improved household flooring is associated with lower odds of enteric and parasitic infections in low- and middle-income countries: A systematic review and meta-analysis

**DOI:** 10.1371/journal.pgph.0002631

**Published:** 2023-12-01

**Authors:** Hugo Legge, Rachel L. Pullan, Benn Sartorius

**Affiliations:** 1 Department of Disease Control, London School of Hygiene & Tropical Medicine, London, United Kingdom; 2 School of Public Health, Faculty of Medicine, The University of Queensland, Brisbane, Queensland, Australia; 3 Centre for Tropical Medicine and Global Health, Nuffield Department of Medicine, University of Oxford, Oxford, United Kingdom; 4 Department of Health Metric Sciences, University of Washington, Seattle, Washington, United States of America; CSIR-Indian Institute of Chemcial Technology, INDIA

## Abstract

Enteric and parasitic infections such as soil-transmitted helminths cause considerable mortality and morbidity in low- and middle-income settings. Earthen household floors are common in many of these settings and could serve as a reservoir for enteric and parasitic pathogens, which can easily be transmitted to new hosts through direct or indirect contact. We conducted a systematic review and meta-analysis to establish whether and to what extent improved household floors decrease the odds of enteric and parasitic infections among occupants compared with occupants living in households with unimproved floors. Following the PRISMA guidelines, we comprehensively searched four electronic databases for studies in low- and middle-income settings measuring household flooring as an exposure and self-reported diarrhoea or any type of enteric or intestinal-parasitic infection as an outcome. Metadata from eligible studies were extracted and transposed on to a study database before being imported into the R software platform for analysis. Study quality was assessed using an adapted version of the Newcastle-Ottawa Quality Assessment Scale. In total 110 studies were eligible for inclusion in the systematic review, of which 65 were eligible for inclusion in the meta-analysis after applying study quality cut-offs. Random-effects meta-analysis suggested that households with improved floors had 0.75 times (95CI: 0.67–0.83) the odds of infection with any type of enteric or parasitic infection compared with household with unimproved floors. Improved floors gave a pooled protective OR of 0.68 (95CI: 0.58–0.8) for helminthic infections and 0.82 OR (95CI: 0.75–0.9) for bacterial or protozoan infections. Overall study quality was poor and there is an urgent need for high-quality experimental studies investigating this relationship. Nevertheless, this study indicates that household flooring may meaningfully contribute towards a substantial portion of the burden of disease for enteric and parasitic infections in low- and middle-income settings.

## Background

Enteric and parasitic infections remain amongst the most common diseases in low- and middle-income settings [1, 2]. Sub-Saharan Africa alone experienced an estimated 330,000 child deaths in 2015 due to diarrhoea-related illness [3]. Known risk factors for enteric infections and parasitic infections such as soil-transmitted helminths include inadequate access to basic levels of water, sanitation and hygiene (WASH), the absence or low coverage of vaccination or preventative chemotherapy programmes, and lower socio-economic status [4].

Ensuring community-wide access to at least basic levels of WASH is a core component of disease control programmes targeting soil-transmitted helminths and diarrhoeal diseases [5]. These interventions aim to control infection risk through reducing exposure to pathogens in the domestic environment. Observational studies typically indicate a relationship between household access to basic WASH services and reduced rates of enteric and parasitic infections and diarrhoea. However, recent large-scale, high-fidelity randomised controlled trials in Bangladesh, Zimbabwe, and Kenya evaluating the impact of different combinations of WASH interventions on childhood diarrhoea and stunting demonstrated either a modest effect or no effect at all on the incidence of diarrhoea [6–8]. These findings suggest that residual pathways for transmission of enteric infections can persist even when access to basic WASH services is ensured.

Hypothosised alternative pathways for infection include contaminated food [9–11], fomites [12], and the presence of animal faeces within the domestic environment [13]. The role of domestic floors, on which pre-school aged children spend a large proportion of their time, is increasingly being investigated as a potentially critical domain on which enteric pathogens are surviving and being transmitted to new hosts [14]. Household floors made from rudimentary or natural materials including sand, soil, clay, and animal dung are of particular interest as they can be difficult to clean and may remain damp, presenting a convenient surface on which pathogens can survive and proliferate. Studies that have conducted environmental sampling from within homes with these floor types have found reservoirs of bacterial, protozoan and parasitic pathogens [15–17].

Despite rapid improvements in the number of houses being built with improved materials across the globe, 12% of households in Latin America and the Caribbean and over half of all houses in Sub-Saharan Africa are made from unimproved materials [18, 19]. Given these vast numbers there is an urgent need to review the available evidence on the relationship between household flooring and enteric and parasitic infections. Despite an absence of experimental studies there is a significant body of observational literature examining this link. This systematic review and meta-analysis aims to establish whether, and to quantify to what extent, domestic flooring acts as an independent risk factor for enteric and parasitic infections in low- and middle-income countries.

## Methods

### Search strategy and screening

The study is in accordance with the Preferred Reporting Items for Systematic Review and Meta-analysis (PRISMA) guidelines. The review protocol has been published [20] and is registered in PROSPERO (CRD42019156437). The PRISMA checklist is available in supplementary materials (S1 Checklist).

We searched EMBASE, MEDLINE, Web of Science, and Google Scholar with no language restrictions for articles published from 1980 onwards. Medical Subject Headings (MESH) search terms were used when searching PubMed/Medline. Search terms used included keywords referring to ‘household flooring’, ‘floor’, ‘dirt’, ‘earthen’, ‘cement’, ‘wood’, ‘tile’, ‘concrete’, ‘hard’, ‘solid’, ‘enteric’, ‘diarrhea’, ‘diarrhoea’, ‘soil-transmitted’, ‘helminth’, ‘worm’, ‘intestinal parasite’, ‘giardia’, ‘entamoeba’. Only studies taking place in low- or middle-income countries (LMIC) were eligible for full screening. Studies including child, adult and all-age populations were eligible for inclusion.

The primary outcome was laboratory-confirmed presence of any enteric or parasitic pathogen using microscopy, serology or molecular methods. The secondary outcomes were (1) presence of any type of helminth infection; (2) presence of a hookworm infection; (3) presence of any type of enteric bacterial or protozoan infection; (4) self-reported, caregiver-reported, or clinical record of diarrhoea taking place within the previous two weeks (S1 Table).

The exposure was type of household floor, which we dichotomized as earthen, mud, sand, dirt, clay, soil, sticks, bamboo floors (referred to in this study as “unimproved floors”) versus cement, concrete, tile, ceramic, parquet, vinyl, carpet, wooden floors (referred to in this study as “improved” floors). Studies that compared floor types in groupings incompatible with the above dichotomization were excluded from the meta-analysis component of this study to ensure comparability of exposure measurements between included studies. Wooden floors were included in the “improved” floor category, as most studies that referenced wood grouped it in this way.

The initial database search and article deduplication was carried out by BS in November 2019. follow-up searches and deduplications were conducted in September 2022 and August 2023 by HL to bring results up-to-date. Title and abstract screening were conducted in duplicate by HL and BS with discordant decisions resolved through mutual agreement by reviewers. Full article screening was conducted by HL.

### Data extraction and quality assessment

Data extraction was conducted by HL using a data capture sheet with pre-specified criteria [20]. Where possible, adjusted measures of effect were extracted. If adjusted analysis was not completed, unadjusted measures of effect were taken instead. If no measure of effect was provided, raw numbers were extracted and used to calculate crude odds ratios and 95% confidence intervals. Where additional meta-data were required, authors were contacted directly.

Study quality was assessed by HL using an adapted version of the Newcastle-Ottawa Quality Assessment Scale for observational studies (S1 File). Studies scoring 50% or less of available points were deemed to have a high risk of bias (cross sectional studies: <6 points; cohort and case control studies <8 points) and those scoring above 50% were considered to have a low risk of bias. A quality control check on bias scoring was undertaken by BS on 10% of included studies with concordance of scoring assessed using Cohen’s Kappa coefficient. Discordant scores were resolved by mutual agreement. Only studies with low-risk of bias were retained for the meta-analysis.

### Data analysis and synthesis

Extracted data was pooled using the tidyverse package on the R software platform (version 4.1.3) [21, 22]. Meta regression, forest plots, and funnel plots were performed using the meta package on the R platform [23]. Pooled results were reported using odds ratios and 95% confidence intervals. Studies reporting prevalence ratios were transformed to odds ratios. Longitudinal studies reporting incidence rate ratios were pooled separately with the intention of carrying out a distinct analysis; however, there were insufficient to carry out this separate analysis so these were excluded from the meta-analysis component of the study. Meta regression was undertaken for each outcome using random-effects models to account for heterogeneity between studies.

Heterogeneity was assessed using the I2 statistic with the following classifications applied: 0% to 40%: might not be important; 30% to 60%: may represent moderate heterogeneity; 50% to 75%: may represent substantial heterogeneity; 75% to 100%: considerable heterogeneity [24]. Publication bias was assessed by examining funnel plots and more formally using Egger’s test statistic.

## Results

### Description of studies

**Fig 1:**
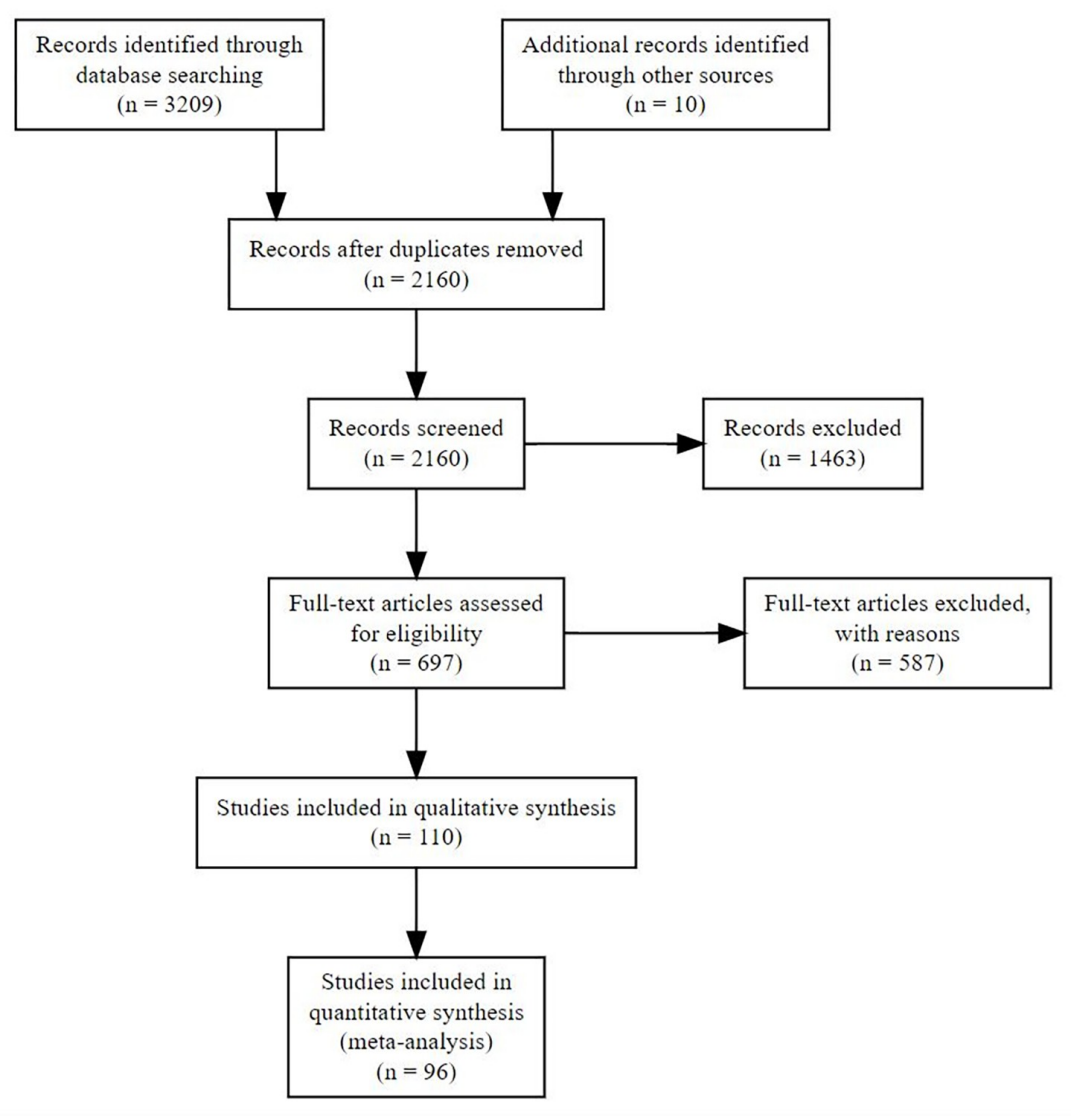
PRISMA flow diagram.

In total, searches returned 3209 articles, of which 1049 were identified as being duplicates and removed. The titles and abstracts of the remaining 2160 articles were screened, of which 697 were identified as being suitable for full article screening. Following article screening 110 [25–133] studies were marked as being eligible for inclusion in the qualitative synthesis (extracted study data is available in supplementary materials; S1 Data). Due to the insufficient number of studies reporting incidence risk ratios or hazard ratios (n = 5) [127–131] these studies were excluded from the meta-analysis component of this study. A further 10 studies included in the qualitative synthesis were excluded from the meta-analysis due to incompatible exposure groupings (grouping wooden floors with earthen and other unimproved floor types) [28, 42, 45, 53, 71, 72, 98, 103, 117, 125]. We contacted 45 authors requesting additional information, of which 11 responded. Based on the information provided nine studies became eligible for inclusion. The final number of studies eligible for inclusion in the meta-analysis was 96, containing a total of 144 separate analyses (Fig 1).

**Fig 2:**
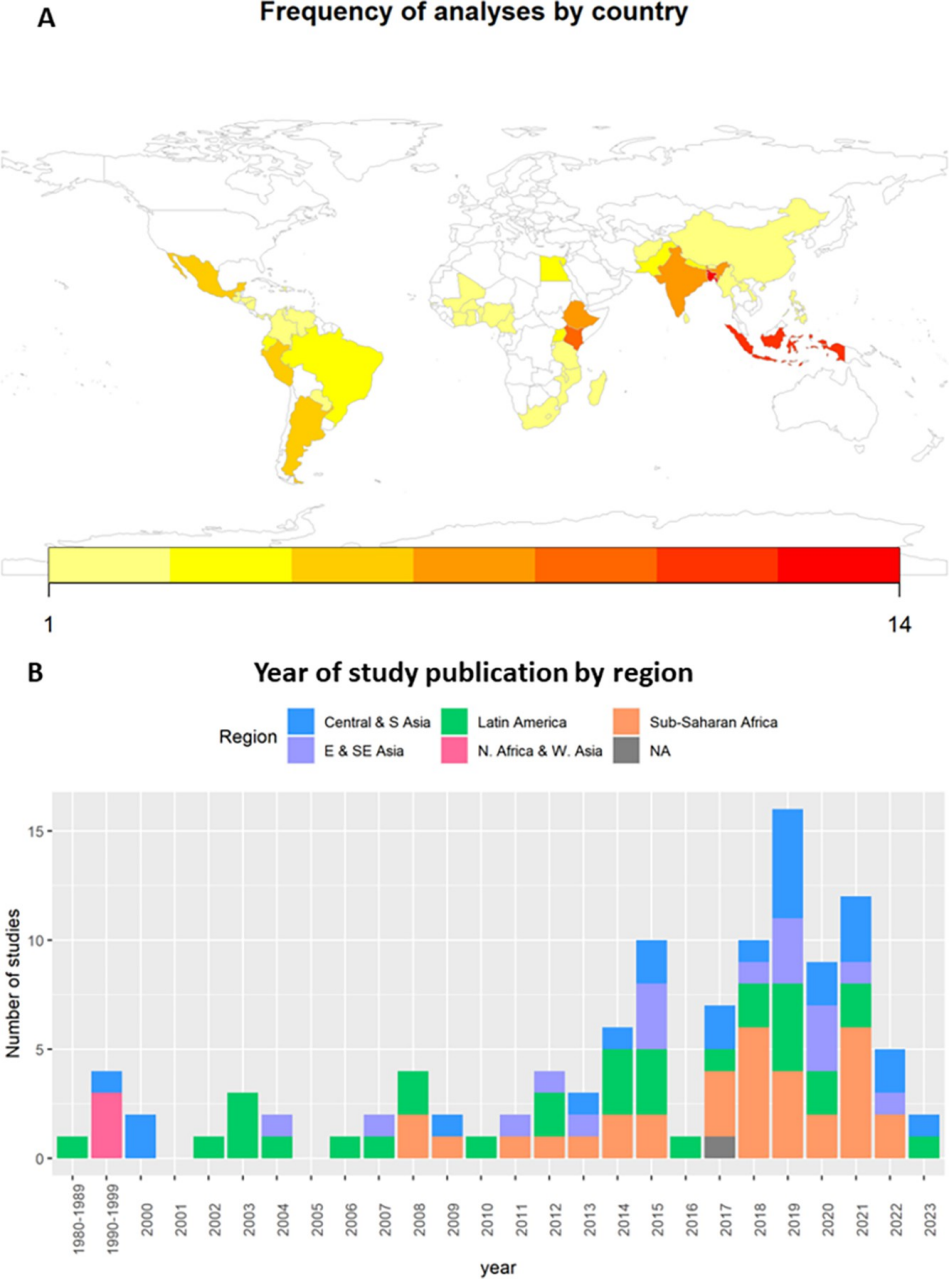
Study metadata. A) frequency of analyses by country, and B) year of study publication by SDG region. Map was created using R Project for Statistical Computing v. 3.3.1 (https://www.R-project.org) and packages rworldmap v. 1.3–6 (https://cran.r-project.org/web/packages/rworldmap/index.html).

All reviewed studies were observational; of which the majority were cross-sectional studies (94/110), followed by cohort studies (10/110) and case-control studies (6/110). Most studies were conducted in either Sub-Saharan Africa (33/110) or Latin America (32/110). Central and South Asia had 24 studies, East and Southeast Asia had 17 studies, and North Africa and West Asia had 3 studies (Fig 2).

### Qualitative synthesis—Categorisation of household flooring

Studies mostly conceptualised flooring dichotomously (104/110) with households grouped according to what studies considered to be improved versus unimproved types of flooring. The majority of studies (87/110) listed the specific flooring materials included in each group (e.g. cement, wood, earthen). Some studies (23/110) provided labels for their flooring categories (e.g. covered, sealed, man-made, natural). Of these 12/23 defined the material types that fit into their categories and 11/23 provided no information on what materials were included within the categories (S2 Table). While category names varied between studies, the specific flooring materials that were grouped together were mostly uniform. In studies where they were referenced, clay, dirt, dung, sawdust, straw, earth, sand and soil floor types were always included in the same grouping. Likewise, in studies where they were referenced, brick, carpet, cement, ceramic tiles, concrete and vinyl were always included in the same grouping. There was more heterogeneity in how bamboo and other wood-based floor types (planks/sticks/timber/wood) were classified. For wood-based floors 12/21 studies grouped them with cement or tile floors, 4/21 studies grouped them with soil-based floors, and 5/21 assigned them to a third “rudimentary” category that also included bamboo and stone. Out of the seven studies that referenced bamboo as a flooring material, four grouped it with soil-based floors and three assigned it to the “rudimentary” category.

Most studies (102/110) did not provide detail on the area within the home that was evaluated to determine the overall domestic floor type. Of those that provided clarification, two classified a dwelling to have an earthen floor if *any* part of the dwelling had an earthen floor; one classified dwellings to have an improved floor if at least one part of the dwelling had a floor material that was deemed to be improved; one used the floor type of the bedroom as a proxy-indicator for the whole dwelling; one specified that whatever was the most common flooring material should be used; and one specified that the categorisation should be dwellings with exclusively improved floors VS exclusively unimproved floors. Studies that used data from Demographic Health surveys (DHS) or Multiple-Indicator Cluster Surveys (MICS) used the most common floor type as a proxy for overall flooring type of the dwelling, but did not necessarily explicitly state this within the manuscript text.

### Meta-analysis–Study populations, exposure, and outcomes

The majority of analyses eligible for inclusion in the meta-regression were conducted exclusively with children <18 years of age (100/144). The remaining 44/144 analyses were conducted either exclusively with adults or with populations comprised of both adults and children. Among the 144 analyses eligible for inclusion, 66 were categorized as being at high risk of bias, while 78 were deemed low risk. In total 69/144 analyses were adjusted, while among studies categorized as being at low risk of bias, 63/78 were adjusted. Cement floors were the most common flooring material to be explicitly referenced among improved flooring materials (96/144) followed by wood floors (36/144). Among unimproved floors, earthen (also including dirt, soil, and sand) was referenced in the great majority of analyses (119/144) (S1 Fig).

Pathogen-specific infections were identified as outcomes in 126/144 of the analyses eligible for inclusion in the meta regression (S3 Table). The remaining 18 analyses measured diarrhoea as an outcome. The majority of studies conducted analyses using a singular pathogen infection as an outcome (78/126), while 48/126 measured the presence of multiple pathogens to denote the presence of a generic infection. Overall, helminth species were specified in 95/126 analyses, with 60/126 analyses measuring *A*. *lumbricoides* infections and 46/126 measuring hookworms. Protozoan and bacterial infections were recorded as outcomes in 48/126 studies, with giardia infections measured in 26 analyses and cryptosporidium measured in 14. Viral infections were only recorded in two analysis and these were grouped with bacterial, helminthic, and protozoan infections to denote presence of a generic enteric infection.

### Meta-analysis–Model results

**Fig 3:**
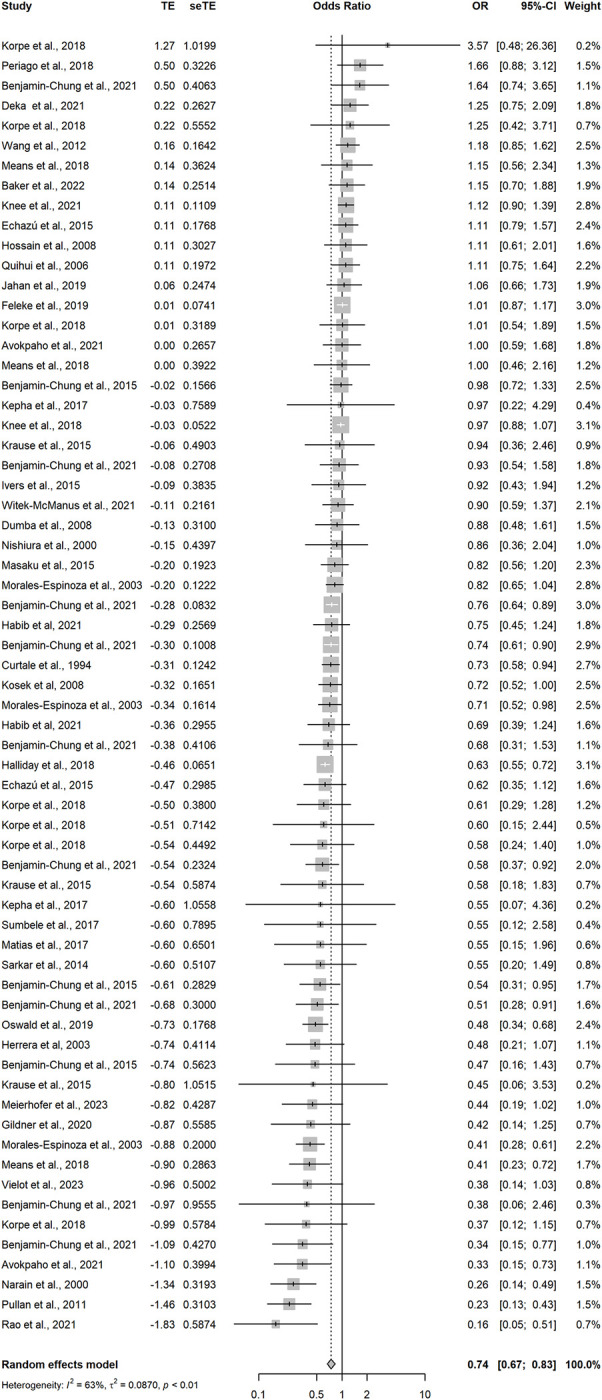
Pooled effect of an improved household floor (VS unimproved floor) on odds of any type of enteric or parasitic infection in any age group (low risk of bias studies only).

**Fig 4:**
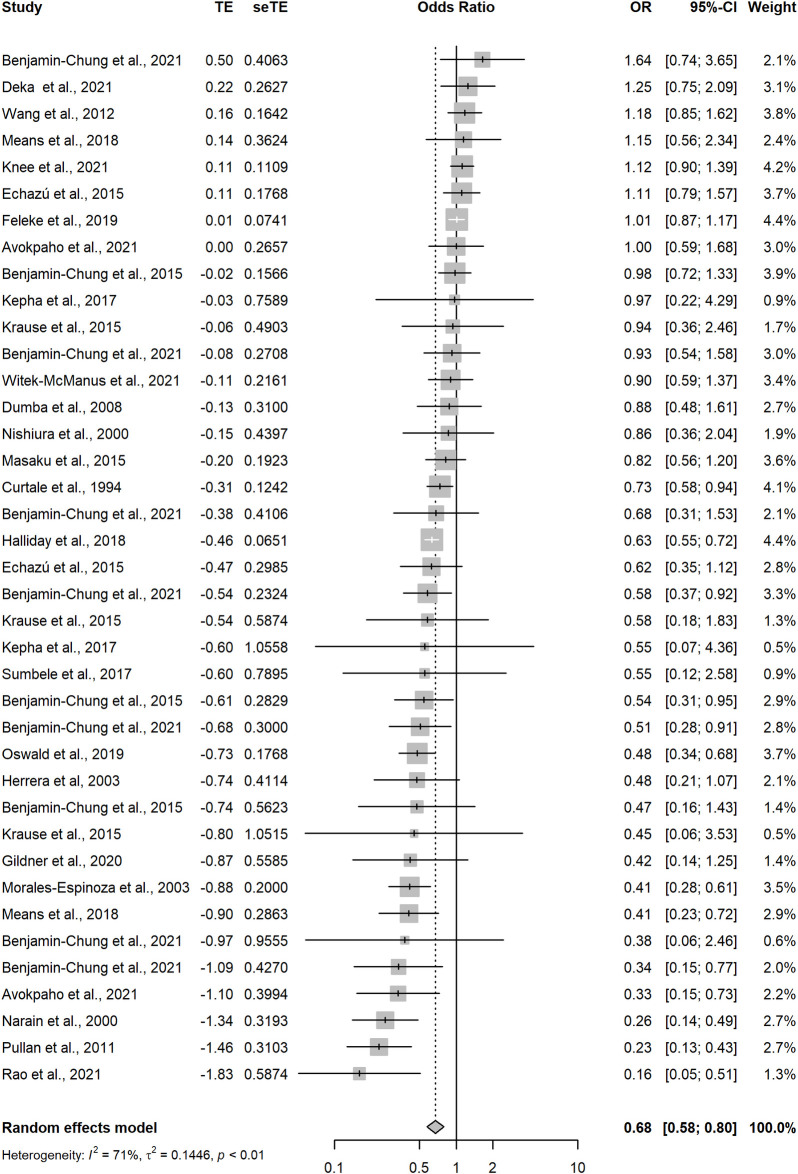
Pooled effect of an improved household floor (VS unimproved floor) on odds of any type of helminthic infection in any age group (low risk of bias studies only).

**Fig 5:**
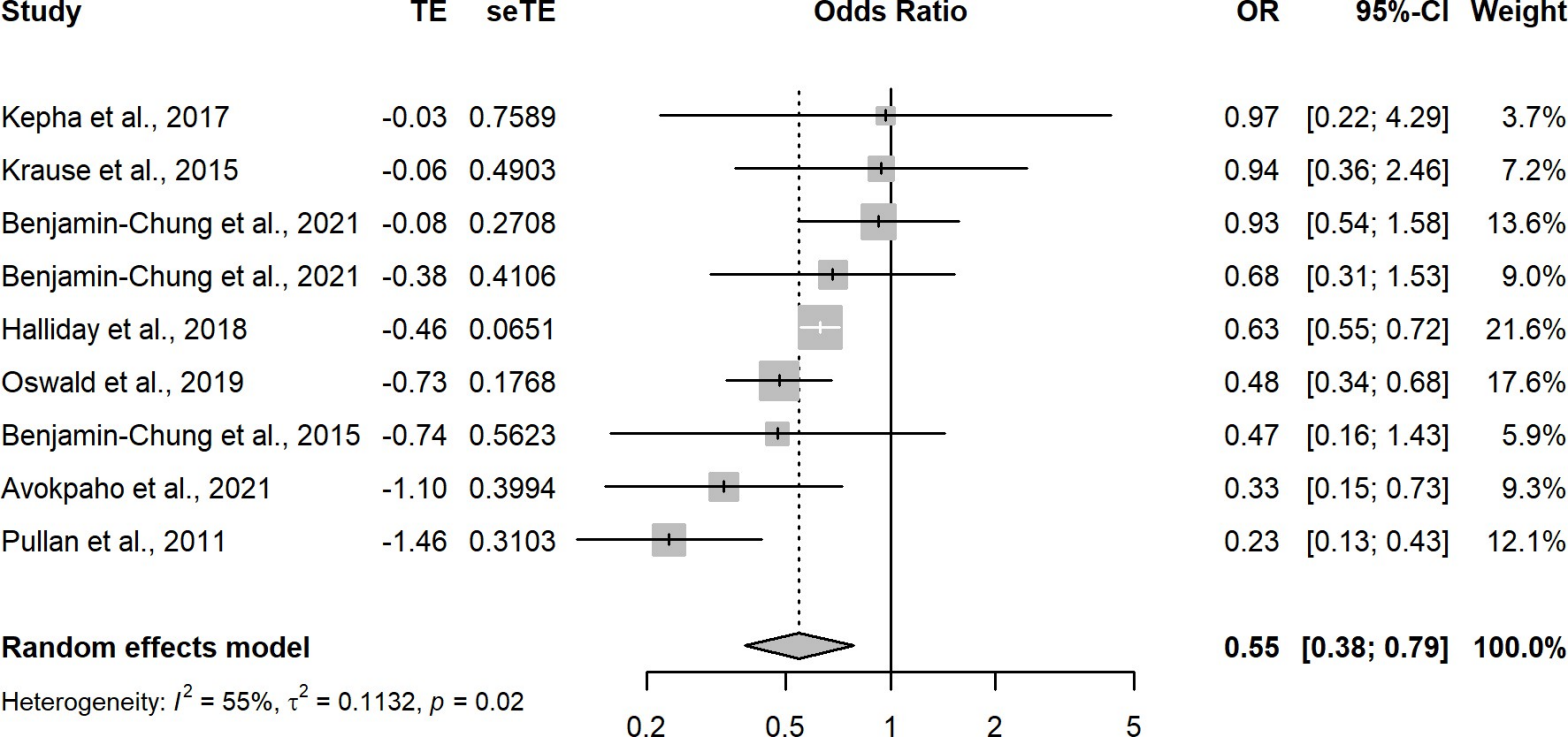
Pooled effect of an improved household floor (VS unimproved floor) on odds of hookworm infections in any age group (low risk of bias studies only).

**Fig 6:**
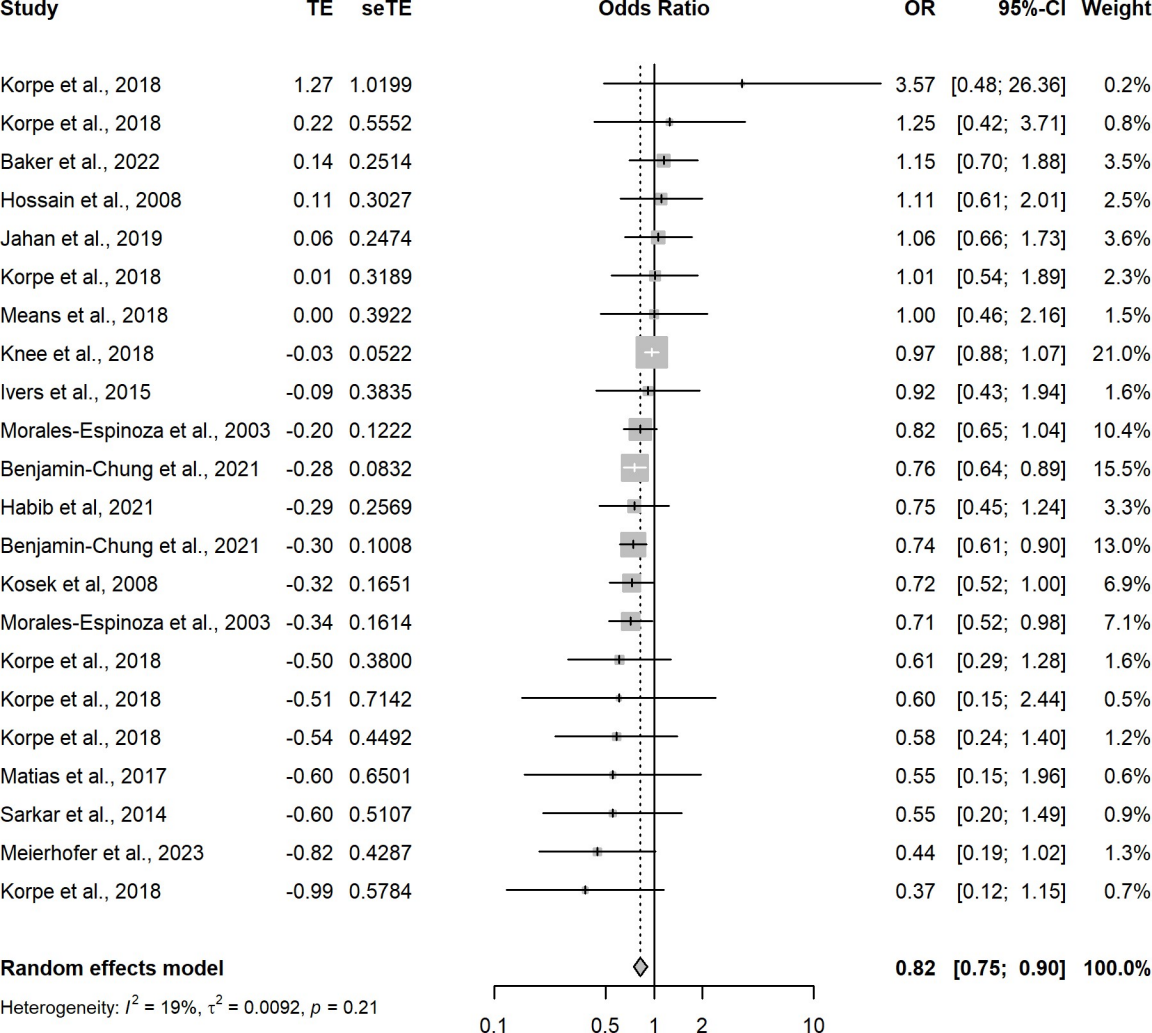
Pooled effect of an improved household floor (VS unimproved floor) on odds of any type of bacterial or protozoan infection in any age group (low risk of bias studies only).

**Fig 7:**
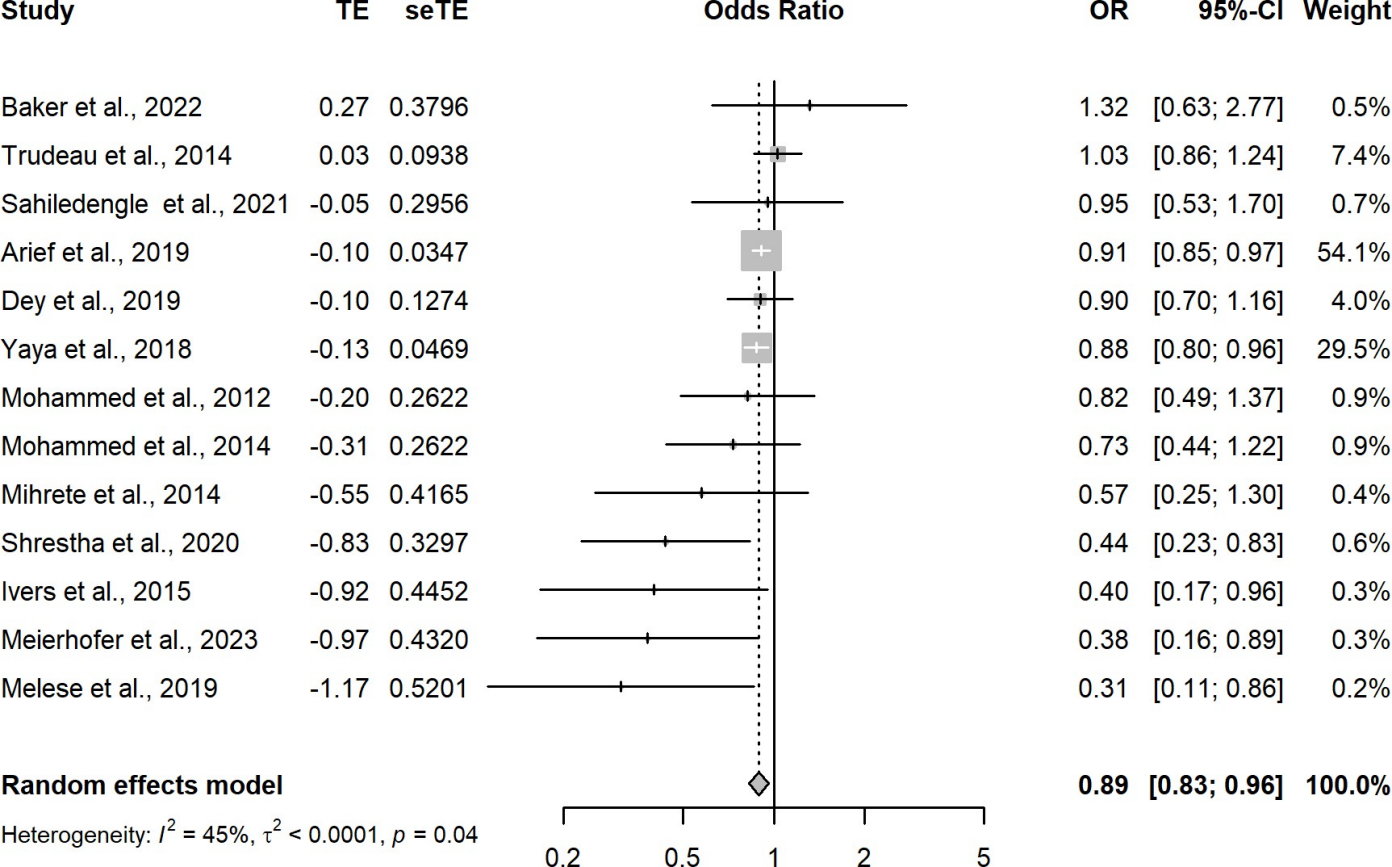
Pooled effect of an improved household floor (VS unimproved floor) on odds of self-reported diarrhoea among any age group (low risk of bias studies only).

Random-effects meta-regression of the 65 analyses with a low risk of bias that measured the association between improved household flooring and presence of at least one enteric or parasitic infection resulted in a pooled OR of 0.74 (95CI: 0.67–0.83) (Fig 3 and Table 1). This indicates that individuals with an improved household floor had 0.74 the odds of having a parasitic or enteric infection compared with individuals living in a dwelling with an unimproved floor. The *I*^2^ was 63%—indicating possible substantial heterogeneity in effect estimates between studies. A model including analyses that measured only helminthic infections (n = 39) produced a pooled OR of 0.68 (95CI: 0.58–0.80) with an *I*^2^ of 71%, again indicating possible substantial heterogeneity in effect estimates between studies (Fig 4). Hookworm-only, low-risk of bias analyses (n = 9) produced a pooled OR of 0.53 (95CI: 0.36–0.77) with an *I*^2^ 55% (Fig 5). Sub-analysis of analyses measuring only bacteria and/or protozoan infections (n = 22) resulted in a pooled OR of 0.82 (95CI: 0.75–0.90) and an *I*^2^ of 19% indicating low-levels of heterogeneity between analyses (Fig 6). Analyses that included diarrhoea as an outcome (n = 13) had a pooled OR of 0.89 (95CI:0.83–0.96) and an *I*^2^ of 45% (Fig 7).

**Table 1:** Random-effects meta-regression model outputs (studies with low-risk of bias only) showing pooled effect of improved flooring (versus unimproved flooring) on outcomes of interest.

Outcome grouping	Region	N*	OR	Lower 95ci	Upper 95ci	I2	Egger p value
All pathogens	All regions	65	0.74	0.67	0.83	0.63	0.02
Diarrhoea exclusive	All regions	13	0.89	0.83	0.96	0.45	0.04
Helminth exclusive	All regions	39	0.68	0.58	0.80	0.71	0.07
Hookworm exclusive	All regions	9	0.55	0.38	0.79	0.55	NA^#^
Protozoa or bacteria exclusive	All regions	22	0.82	0.75	0.90	0.19	0.25
All pathogens	Central & S Asia	18	0.73	0.57	0.92	0.52	0.43
All pathogens	Latin America	18	0.71	0.56	0.89	0.54	0.15
All pathogens	SS Africa	27	0.75	0.64	0.89	0.72	0.22

*Including only studies with low risk of bias. Table with outputs from models including (1) all studies and (2) studies with low and medium risk of bias can be found in S4 Table in the supplementary materials

^#^Too few studies (<10) in this analyses grouping to perform egger test.

A separate sub-group analysis conducted by region showed negligible difference on the pooled effect estimates of household improved flooring on any enteric or parasitic infection between regions (Sub-Saharan Africa = 0.75 (95CI: 0.64–0.87) / Central and East Asia = 0.73 (95CI: 0.57–0.92) / Latin America = 0.71 (95CI: 0.56–0.89)). Funnel plots were also run for each outcome grouping and are presented in the supplementary materials (S2–S6 Figs).

## Discussion

This study is the first of its kind to undertake a systematic review and meta-analysis examining the association between household flooring and enteric and parasitic infections. The meta-analysis demonstrated that in low- and middle-income settings improved household flooring is protective against enteric and parasitic infections when compared with unimproved household flooring. Improved flooring was found to be significantly protective against generic helminthic infections, hookworm-specific infections, generic enteric bacterial and protozoan infections, and diarrhoea. The largest pooled effect estimates were observed when including studies measuring hookworm infections or generic helminthic infections as outcomes. A sub-group analysis by region showed no meaningful difference in the protective effect of improved flooring between Sub-Saharan Africa, Central and East Asia, and Latin America. However, given that the protective effect of improved household flooring varied according to pathogen type, the epidemiological profile of a country or region will be an important consideration when evaluating the role of flooring in facilitating transmission of enteric and parasitic infections.

A limitation of this systematic review and meta-analysis is that it relies exclusively on results from observational studies, as to date, no experimental research evaluating the relationship between flooring and enteric or parasitic infections has been published in the literature. Results should therefore be treated with some caution as they are vulnerable to confounding and publication bias. To mitigate this risk, the analysis presented in this paper adopted a high threshold for study inclusion based on risk of bias score. Nevertheless, it is possible that residual confounding, particularly in relation to the role of socio-economic status (which could be independently associated with both exposure and outcome) may have substantially influenced these results, especially in studies that were not adjusted for these factors.

Results from this meta-analysis support findings from previous environmental sampling studies that have found high levels of bacterial contamination [15], as well as viruses, protozoa and helminths on earthen household floors [16, 17]. Among household members, infants and young children are potentially more likely to be exposed to these pathogens as they explore environments with their hands and commonly perform hand-to-mouth actions [14, 134, 135]. If infected, children are also more likely to experience severe disease compared with adults [1]. Future research should focus on conducting high-quality, randomised experimental studies that can evaluate the impact of improved flooring on child and adult health, isolated from potential confounders such as socio-economic status and access to WASH. Outcomes of interest could include enteric infections, soil-transmitted helminthiasis, and diarrhoea. Future research could also examine the persistence of different pathogens on improved floors such as cement and tiled surfaces, as only a handful of studies have so far examined this [15, 17]. Beyond physical health, future studies could also explore whether household flooring affects the psychological wellbeing of occupants, including both adults and children. and whether broader non-health outcomes such as economic activity and educational performance are linked with household flooring type.

There was some evidence for publication bias in the pooled model. This could be in part explained by the fact that many of the included studies were not designed to examine household flooring as a primary exposure of interest, but instead as part of a wider suite of covariates in a generic risk factor analysis. As a result, in some studies where household flooring was found to be insufficiently associated with the outcome it was dropped from the final multivariate model and its measure of effect was not reported, despite its initial inclusion. This phenomenon makes the availability of studies reporting a significant association between household flooring and health outcomes more likely when compared to studies that report no effect. To address this, authors were contacted to provide the adjusted measure of effect, and if no response was provided then the univariate measure of effect was used and a higher risk of bias score was applied to the study.

There was heterogeneity in how studies categorized and reported household flooring. Primarily this related to how wood and bamboo floors were classified, with some studies including them with cement-based and other “improved” floor types and other studies grouping them with earthen and other “unimproved” floor types. For future studies, adoption of a standardized classification system for floors would improve inter-study comparability. Currently, the DHS and MICS question modules are aligned on how to measure household flooring, and as such offer a validated and widely-adopted criteria for measuring floors that future studies should consider adopting. However, using a one-size-fits-all method for measuring and categorizing flooring comes at the risk of jeopardizing the internal validity of studies, as local variations in flooring types may not be captured by the DHS/MICS question.

Findings from this systematic review and meta-analysis indicate that household flooring should be considered an important domain of transmission for enteric and parasitic infections in low- and middle-income countries. The substantial number of households that reside in homes built with unimproved materials in low- and middle-income countries suggest that the collective cost in morbidity and mortality due to unimproved flooring could be considerable. However, the quality of available evidence is weak and as such there is an urgent need for high-quality experimental studies investigating the relationship between flooring and enteric and parasitic infections.

## Supporting information

S1 Checklist PRISMA checklist. (DOCX)

S1 Table Outcome categorisation. (DOCX)

S2 Table Flooring categories with their definitions. (DOCX)

S3 Table Study outcomes. (DOCX)

S4 Table Random-effects meta-regression model outputs. (DOCX)

S1 Fig Frequency of flooring materials referenced in studies. (DOCX)

S2 Fig Funnel plot for analyses reporting any type of pathogen infection (low risk of bias only). (DOCX)

S3 Fig Funnel plot for analyses exclusively reporting diarrhoea (low risk of bias only). (DOCX)

S4 Fig Funnel plot for analyses exclusively reporting helminth infections (low risk of bias only). (DOCX)

S5 Fig Funnel plot for analyses exclusively reporting hookworm infections (low risk of bias only). (DOCX)

S6 Fig Funnel plot analyses exclusively reporting bacterial or protozoan infections (low risk of bias only). (DOCX)

S1 File Newcastle-Ottawa study quality scale. (DOCX)

S1 Data Study data. (CSV)

S2 Data Data dictionary. (CSV)
