# Supplementary material for: Improved household flooring is associated with lower odds of enteric and parasitic infections in low- and middle-income countries: A systematic review and meta-analysis
Source: PLOS Glob Public Health. 2023 Dec 1;3(12):e0002631. doi: 10.1371/journal.pgph.0002631 (PMC10691699; doi:10.1371/journal.pgph.0002631)
Supplement: S1 Table — (DOCX) [file pgph.0002631.s002.docx]

S1 Table. Outcome categorisation

| Meta-analysis outcome grouping | Inclusion criteria | Exclusion criteria |
| --- | --- | --- |
| Pooled enteric / parasitic infections | Any analysis that reports laboratory-confirmed presence of one or more enteric bacterial, protozoan, viral, or parasitic infection(s) | None |
| Pooled helminthic infections | Any analysis that reports laboratory-confirmed presence of one or more helminthic infection(s) | Presence of one or more non-helminthic (i.e. bacterial, protozoan, viral) infection(s) |
| Hookworm infections | Any analysis that reports laboratory-confirmed presence of either (or both) hookworm species | Presence of one or more non-hookworm infection(s) |
| Pooled bacterial/protozoan infections | Any analysis that reports laboratory-confirmed presence of one or more protozoan or bacterial infection(s) | Presence of one or more non-bacterial or protozoan (i.e. helminthic or viral) infection(s) |
| Diarrhoea total | Any analysis that includes clinical or self-report measures of diarrhoea (any severity) | None |
