# Supplementary material for: Improved household flooring is associated with lower odds of enteric and parasitic infections in low- and middle-income countries: A systematic review and meta-analysis
Source: PLOS Glob Public Health. 2023 Dec 1;3(12):e0002631. doi: 10.1371/journal.pgph.0002631 (PMC10691699; doi:10.1371/journal.pgph.0002631)
Supplement: S2 Table — (DOCX) [file pgph.0002631.s003.docx]

S2 Table. Flooring categories with their definitions

| Flooring category | No of studies using category | Description of category | No of studies using definition of category |
| --- | --- | --- | --- |
| Unsafe flooring | 1 | Earthen | 1 |
| Unsealed floor | 2 | No definition provided | 2 |
| Natural | 6 | No definition provided | 3 |
|  |  | Sand, dung, straw, and sawdust | 1 |
|  |  | earth, dung, palm or bamboo and stone | 1 |
|  |  | Earth, sand, dung | 1 |
| Unfinished floor | 2 | No definition provided | 1 |
|  |  | Earth/bamboo | 1 |
| No floor present | 2 | No definition provided | 2 |
| Not covered | 2 | No definition provided | 1 |
|  |  | Packed dirt floor | 1 |
| Unimproved | 4 | Dirt | 3 |
|  |  | No definition provided | 1 |
|  | | | |
| Rudimentary | 2 | wood planks, palm, bamboo | 2 |
|  | | | |
| Finished flooring | 5 | No definition provided | 1 |
|  |  | wood, vinyl, ceramic tiles, cement, carpet, and brick | 1 |
|  |  | Parquet, polished wood, vinyl, asphalt strips, plastic tile, cement, carpet | 1 |
|  |  | Cement,wood | 1 |
|  |  | wood, tile, concrete | 1 |
| Covered | 3 | No definition provided | 1 |
|  |  | Not earthen | 1 |
|  |  | Some sort of flooring material such as concrete | 1 |
| Safe Flooring | 1 | Cement, iron, timber, stone | 1 |
| Sealed floor | 2 | No definition provided | 2 |
| Floor present | 2 | No definition provided | 2 |
| Improved | 4 | No definition provided | 1 |
|  |  | Bricks or cement | 2 |
|  |  | Cement or wood | 1 |
| Man-made | 4 | No definition provided | 3 |
|  |  | wood, brick, vinyl or asphalt strips, tiles, cement, carpet and polished stone like marble or granite | 1 |
