# Supplementary material for: Improved household flooring is associated with lower odds of enteric and parasitic infections in low- and middle-income countries: A systematic review and meta-analysis
Source: PLOS Glob Public Health. 2023 Dec 1;3(12):e0002631. doi: 10.1371/journal.pgph.0002631 (PMC10691699; doi:10.1371/journal.pgph.0002631)
Supplement: S3 Table — (DOCX) [file pgph.0002631.s004.docx]

S3 Table. Study outcomes

| group | Infection outcomes | Unique analysis of this pathogen | Included within helminth-exclusive analysis | Included within  Bacteria / Protozoa-exclusive analysis | Included within grouped analysis of helminth / bacteria / protozoa /virus | Total studies referencing this outcome |
| --- | --- | --- | --- | --- | --- | --- |
| Diarrhoea | diarrhoea | 18 | 0 | 0 | 0 | 18 |
| Helminth | a_lumbricoides | 20 | 31 | 0 | 15 | 66 |
|  | a_duodenale | 14 | 27 | 0 | 8 | 49 |
|  | n_americanus | 16 | 28 | 0 | 8 | 52 |
|  | t_trichiura | 9 | 31 | 0 | 13 | 53 |
|  | strongyloides | 3 | 4 | 0 | 3 | 10 |
|  | e_vermicularis | 0 | 3 | 0 | 11 | 14 |
|  | h_nana | 1 | 5 | 0 | 11 | 17 |
|  | a_ceylanicum | 1 | 0 | 0 | 0 | 1 |
|  | t_solium | 0 | 1 | 0 | 0 | 1 |
|  | t_saginata | 0 | 1 | 0 | 0 | 1 |
|  | s_mansoni | 3 | 0 | 0 | 2 | 5 |
| Bacteria/  protozoa | giardia | 10 | 0 | 2 | 15 | 27 |
|  | cholera | 3 | 0 | 1 | 1 | 5 |
|  | cryptosporidium | 10 | 0 | 1 | 3 | 14 |
|  | e_histolytica | 2 | 0 | 1 | 7 | 10 |
|  | shigella | 2 | 0 | 1 | 1 | 4 |
|  | toxoplasmosis | 1 | 0 | 0 | 0 | 1 |
|  | blastocystis | 1 | 0 | 1 | 4 | 6 |
|  | c_mesnili | 0 | 0 | 0 | 4 | 4 |
|  | i_butschlii | 0 | 0 | 0 | 4 | 4 |
|  | c_cayetanensis | 0 | 0 | 0 | 2 | 2 |
|  | e_nana | 0 | 0 | 0 | 7 | 7 |
|  | e_coli | 1 | 0 | 1 | 10 | 12 |
|  | salmonella | 0 | 0 | 1 | 1 | 2 |
|  | campylobacter | 0 | 0 | 1 | 1 | 2 |
|  | e_hominis | 0 | 0 | 0 | 1 | 1 |
|  | y_enterocolitica | 0 | 0 | 0 | 1 | 1 |
| Virus | norovirus | 1 | 0 | 0 | 1 | 2 |
|  | andenovirus | 0 | 0 | 0 | 1 | 1 |
|  | rotavirus | 0 | 0 | 0 | 1 | 1 |
