# Supplementary material for: Improved household flooring is associated with lower odds of enteric and parasitic infections in low- and middle-income countries: A systematic review and meta-analysis
Source: PLOS Glob Public Health. 2023 Dec 1;3(12):e0002631. doi: 10.1371/journal.pgph.0002631 (PMC10691699; doi:10.1371/journal.pgph.0002631)
Supplement: S4 Table — (DOCX) [file pgph.0002631.s005.docx]

S4 Table. Random-effects meta-regression model outputs (all study groupings)

| outcome grouping | Bias categorization | Region | K | OR | Lower  95ci | Upper  95ci | I2 | egger_p_value |
| --- | --- | --- | --- | --- | --- | --- | --- | --- |
| All pathogens | 1. all studies included | All areas | 126 | 0.62 | 0.55 | 0.69 | 0.76 | 0.00 |
| All pathogens | 2. bottom 33% excluded | All areas | 90 | 0.68 | 0.62 | 0.75 | 0.68 | 0.00 |
| All pathogens | 3. bottom 50% excluded | All areas | 65 | 0.74 | 0.67 | 0.83 | 0.63 | 0.03 |
| Diarrhoea excl | 1. all studies included | All areas | 18 | 0.82 | 0.71 | 0.95 | 0.44 | 0.03 |
| Diarrhoea excl | 2. bottom 33% excluded | All areas | 17 | 0.82 | 0.70 | 0.95 | 0.47 | 0.02 |
| Diarrhoea excl | 3. bottom 50% excluded | All areas | 13 | 0.89 | 0.83 | 0.96 | 0.45 | 0.04 |
| Helminth excl | 1. all studies included | All areas | 79 | 0.60 | 0.53 | 0.69 | 0.78 | 0.01 |
| Helminth excl | 2. bottom 33% excluded | All areas | 57 | 0.65 | 0.57 | 0.74 | 0.72 | 0.00 |
| Helminth excl | 3. bottom 50% excluded | All areas | 39 | 0.68 | 0.58 | 0.80 | 0.71 | 0.07 |
| Hookworm excl | 1. all studies included | All areas | 14 | 0.57 | 0.42 | 0.79 | 0.57 | 0.73 |
| Hookworm excl | 2. bottom 33% excluded | All areas | 13 | 0.55 | 0.40 | 0.76 | 0.57 | 0.53 |
| Hookworm excl | 3. bottom 50% excluded | All areas | 9 | 0.55 | 0.38 | 0.79 | 0.55 |  |
| Protazoa excl | 1. all studies included | All areas | 30 | 0.73 | 0.63 | 0.85 | 0.52 | 0.02 |
| Protazoa excl | 2. bottom 33% excluded | All areas | 25 | 0.77 | 0.68 | 0.87 | 0.46 | 0.06 |
| Protazoa excl | 3. bottom 50% excluded | All areas | 22 | 0.82 | 0.75 | 0.90 | 0.19 | 0.25 |
| All pathogens | 3. bottom 50% excluded | Central & S Asia | 18 | 0.73 | 0.57 | 0.92 | 0.52 | 0.44 |
| All pathogens | 3. bottom 50% excluded | Latin America | 18 | 0.71 | 0.56 | 0.89 | 0.54 | 0.16 |
| All pathogens | 3. bottom 50% excluded | SS Africa | 27 | 0.75 | 0.64 | 0.89 | 0.72 | 0.22 |

Rows highlighted green indicate the models used in the main meta-regression analysis.
