# Supplementary figures and images for: Improved household flooring is associated with lower odds of enteric and parasitic infections in low- and middle-income countries: A systematic review and meta-analysis
Source: PLOS Glob Public Health. 2023 Dec 1;3(12):e0002631. doi: 10.1371/journal.pgph.0002631 (PMC10691699; doi:10.1371/journal.pgph.0002631)

S1 Fig. Frequency of flooring materials referenced in studies eligible for the meta-analysis.


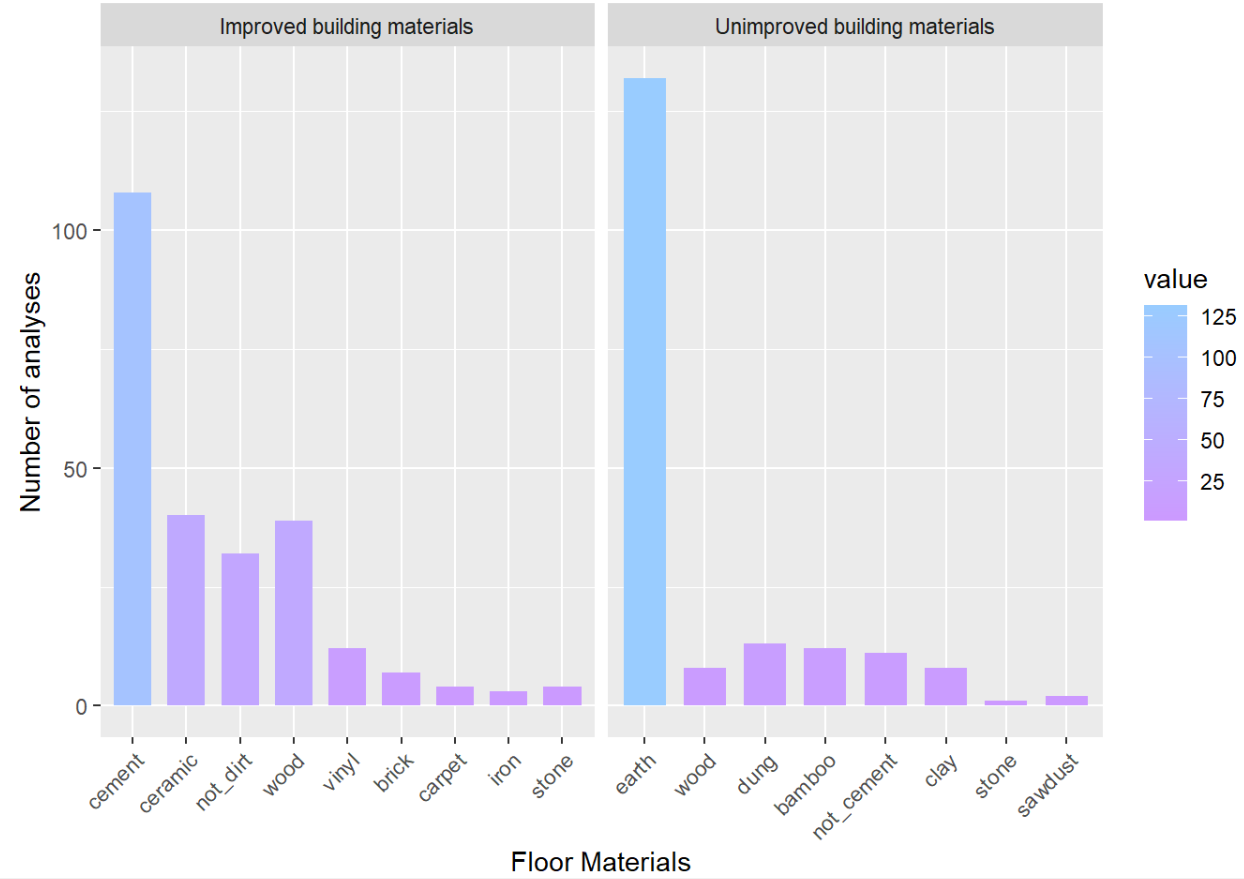

Supplement: S1 Fig — (DOCX) [file pgph.0002631.s006.docx]

S2 Fig. Funnel plot for analyses reporting any type of pathogen infection (low-risk of bias

only) (n=65)


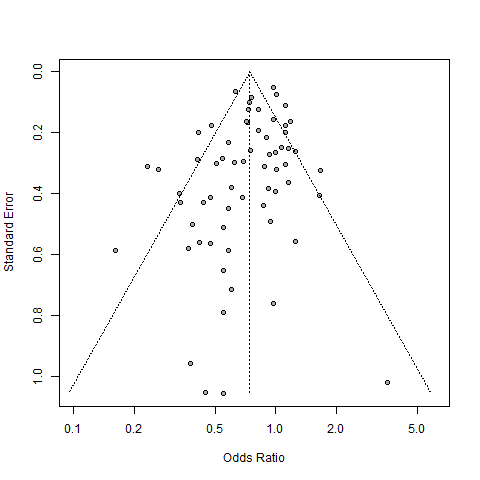

Supplement: S2 Fig — (DOCX) [file pgph.0002631.s007.docx]

S3 Fig. plot for analyses exclusively reporting diarrhoea (low-risk of bias

only) (n=13)


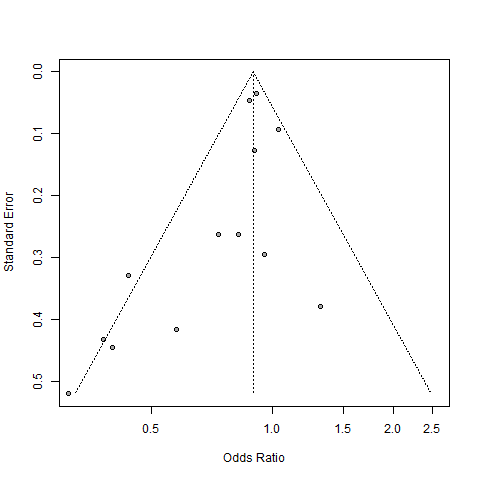

Supplement: S3 Fig — (DOCX) [file pgph.0002631.s008.docx]
