# Supplementary material for: Improved household flooring is associated with lower odds of enteric and parasitic infections in low- and middle-income countries: A systematic review and meta-analysis
Source: PLOS Glob Public Health. 2023 Dec 1;3(12):e0002631. doi: 10.1371/journal.pgph.0002631 (PMC10691699; doi:10.1371/journal.pgph.0002631)

S4 Fig. Funnel plot for analyses exclusively reporting helminth infections (low-risk of bias

only) (n=39)


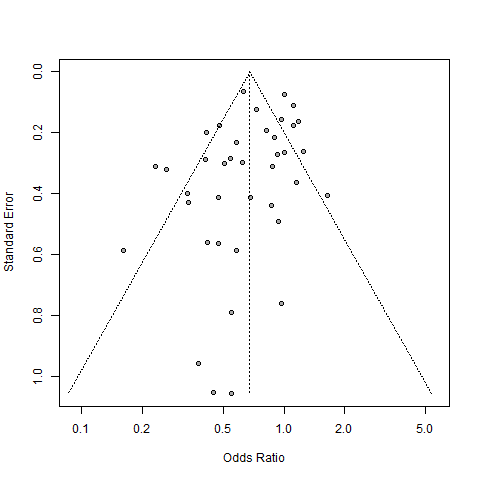

Supplement: S4 Fig — (DOCX) [file pgph.0002631.s009.docx]
